# An Animated Manikin

**Published:** 1891-04

**Authors:** 


### An Animated Manikin.

In the Vienna letter to the *Medical Record*, speaking of the throat-clinics the correspondent says: No notice of throat study here would be complete which ignored the distinguished usefulness of one Frau Gailey, a ward employee in Professor Shrotter's clinic. Though at least sixty and though she has lost one eye, this person exercises a marked fascination over all young students. The charm lies in the remarkable tolerance of instruments and foreign bodies which her larynx has by long practice acquired. Her occupation has thus come to be that of an animated manikin. For one gulden (40 cents) and a glass of wine she permits the student to introduce instruments, place and return foreign bodies, etc., by the hour. So skilled is she that the operator's mistakes are corrected by her hand directing his, till the right spot is reached.

